# Conduct disorder—A sequelae of viral encephalitis

**DOI:** 10.4103/0019-5545.31560

**Published:** 2006

**Authors:** Kamala Deka, Dhrubajyoti Bhuyan, P.K. Chaudhury

**Affiliations:** * Associate Professor, Department of Psychiatry, Assam Medical College and Hospital, Dibrugarh, Assam; ** Postgraduate Student, Department of Psychiatry, Assam Medical College and Hospital, Dibrugarh, Assam; *** Professor and Head, Department of Psychiatry, Assam Medical College and Hospital, Dibrugarh, Assam

**Keywords:** Conduct disorder, viral encephalitis

## Abstract

An 11-year-old girl presented with a behavioural problem of 2 years' duration, which developed following an attack of viral encephalitis. Her behavioural changes had manifested as conduct disorder and were treated with pharmacotherapy as well as behavioural therapy.

## INTROUCTION

Conduct disorder is a disruptive behaviour disorder in children characterized by repetitive pattern of behaviour that violates the rights of others or major social rules.^1^,^2^ There is no pathognomonic or essential symptom to diagnose conduct disorder, rather a range of acts define the condition by their numbers, severity and persistence for a duration of at least 12 months. The behaviour, viewed as symptoms of the conduct disorder, is grouped into four categories: (i) physical aggression or threats of harm to people or animals; (ii) destruction of property; (iii) acts of deceitfulness or theft; and (iv) serious violation of age-appropriate rules.

Encephalitis—inflammation of the brain—is a common disorder in the northeastern part of India. It is caused by viruses of various types of which Japanese B encephalitis is the well-known infective agent. Encephalitis ranges from mild to severe and may result in permanent neurological damage and even death. But in some children and young adults it may present with psychiatric problems such as personality and temperamental changes,^3^–^6^ which usually are hyperactivity and impulsive and antisocial behaviours.^3^

In this patient, behavioural changes following encephalitis were becoming a matter of concern not only to her parents but also to others.

## THE CASE

An 11-year-old girl, who was in class IV and belonged to the lower socioeconomic class (Kuppuswami scale) from village Aradhol (district Dhemaji), was presented to the psychiatry OPD of Assam Medical College on 20 December 2004 with a 2-year history of behavioural abnormalities in the form of repeatedly running away from home, frequent lying, stealing things, and killing domestic birds and animals. The girl, who was reported to be absolutely normal and functioning normally in all areas including her scholastic performance, developed behavioural problems soon after having continuous fever for 15 days along with altered sensorium. She had no history of seizure and was diagnosed by a paediatrician as having viral encephalitis (VE), which was endemic at that time in that locality. As there was no facility for sophisticated investigations, the paediatrician decided not to investigate for confirmation of diagnosis when the clinical picture and the history were suggestive of VE. She was then treated in a local nursing home with steroids and antibiotics (injection ceftriaxone 500 mg i.v., b.i.d.) for a period of 15 days. However, no anti-epileptic or anti-malarial or anti-viral drugs were prescribed at that time. She recovered completely from VE without any residual physical or neurological symptoms.

Soon after the attack of VE, the girl gradually became more and more irritable, used to get angry at slightest provocation and started using abusive words. She became increasingly restless. After one month of the subsidence of the fever, she resumed going to school, but on many occasions she ran away from the school. She took up the habit of teasing and tormenting her classmates, stealing their tiffin and pulling their hair. Her teachers repeatedly complained to her parents.

Gradually, she started going out of the house, destroying property and eating up meals cooked for others without their permission. She also started stealing things like *chappals* and garments kept outside neighbours' houses. Whenever she was caught red-handed she would apologize but the very next moment repeat the naughty behaviour. She would tell lies frequently. She would run away to distant places and remain away for 10–15 days at a stretch. She would easily fox strangers by saying that she belonged to a very poor family and had a stepmother who tortured and tormented her. Most people would believe her story and would give her shelter. There also she would not stay for long because either they would come to know about her real identity or she would run away after stealing garments or ornaments. Her parents reported 10 such instances when she ran away from home during the past 2 years. Once she stole a 6-month-old baby who was kept in the *verandah* while the mother of the baby was busy in household activities. The baby was recovered the same day with the help of the police from the patient's house.

During the period she also showed aggressive and cruel behaviour towards animals by killing ducks and hens of her own household and also those of her neighbours. At times, her parents had to tie her up inside the house but somehow she managed to escape. Her neighbours also took to beating her for her unwanted behaviour.

She was the third child of a nuclear family. Her parents and other two siblings enjoyed good health. There was no family history of any mental or physical illness. Her parents consulted many faith healers for her problematic behaviour before consulting a local physician who referred the patient to the Assam Medical College, Dibrugarh for psychiatric consultation. She was brought to the psychiatry OPD at the Assam Medical College. Prior to the admission she had never received any kind of psychotropic medication.

The girl with normal developmental milestones and premorbidly ‘easy temperament’ was thoroughly examined physically which revealed no abnormality. Mental status examination revealed a demanding child with relevant coherent but pedantic speech with expansion mood and congruent affect in full range. She was fidgety and behaved over-friendly with the interviewer. Her attention and concentration was ill-sustained but judgement was intact with insight preserved.

Routine examination of the blood, EEG and CT scan were done, which revealed no abnormality. She was of average intelligence (IQ=110). She was provisionally diagnosed as a case of conduct disorder. Carbamazepine 300 mg/day and haloperidol 1.5 mg/day along with behavioural therapy (token economy) were started. She has been followed-up monthly and the 2 follow-ups have shown definite improvement in her behaviour. She has gone out of the house without informing anyone only once in the past 2 months. Though she continues to tell lies, no stealing and cruelty towards animals has been reported so far—which is an encouraging factor for the parents as well as the physicians.

## DISCUSSION

‘Just as we find it hard today to follow up the trend of thought of our scientific predecessors for whom bacteriology and the lore of brain localization did not exist, further generations will hardly be able to appreciate our pre-encephalitic neurological and psychiatric conceptions particularly with regard to so-called functional disturbances…’—Von Economo (1929).^4^

VE is known to present with psychiatric symptoms during its active phase as well as post-encephalitic sequelae.^4^ Even the classic work of Economo^4^ on encephalitis documents a psychotic form of encephalitic presentation. Psychosis, parkinsonism and personality changes are well known sequelae of encephalitic. It is estimated that approximately one-third of patients below 16 years of age develop some form of mental changes following encephalitis lethargica. Behavioural and personality changes are seen mostly in children and young adults.^4^ Hyperactivity and oppositional behaviour in children may be seen following encephalitis. Lishman also describes the typical behavioural changes following encephalitis. According to him, over-activity and antisocial behaviour are common post-encephalitic sequelae.^4^ The behaviour of the girl in this case was definitely a violation of age-appropriate social norms.

Characteristic personality changes following encephalitis are emotional lability with destructive, abusive behaviour, which are hard to control. Frequently the child appears to be aware enouh of these changes in him/her to apologize repeatedly but soon afterwards he/she is compelled to repeat the behaviour. In case of this girl too, emotional labilities were marked with repeated apologies for her behaviour but immediately afterwards she was seen to repeat the same kind of behaviour.

With these typical post-VE behavioural changes which meet the ICD-10 and DSM-IV-TR criteria for conduct disorder, that too in a girl with absolutely normal functioning before the attack of VE, is enough reason to consider her behavioural problem to be a consequence of encephalitis. Whatever may be the frequency of occurrence of this type of behavioural change, it is a reminder of the good research work of some of the great neuropsychiatrists of the past who described the behavioural and temperamental changes that can occur following VE.
